# Chylothorax in a 57-Year-Old Female Patient After a Car Accident

**DOI:** 10.3390/jcm15155877

**Published:** 2026-07-27

**Authors:** Melania Mikołajczyk-Solińska, Bartłomiej Czyżak, Mateusz Zdaniewicz, Andrzej Węgiel, Marlena Gajczak, Tomasz Szpotan, Agata Majos, Jacek Kasznicki

**Affiliations:** 1 Department of Internal Medicine, Diabetology and Clinical Pharmacology, Medical University of Lodz, Pomorska Street 251, 92-213 Lodz, Poland; jacek.kasznicki@umed.lodz.pl; 2 Student Scientific Society of Civilization Diseases, Medical University of Lodz, Pomorska Street 251, 92-213 Lodz, Poland; bartlomiej.czyzak@student.umed.lodz.pl (B.C.); mateusz.zdaniewicz@student.umed.lodz.pl (M.Z.); andrzej.wegiel@umed.lodz.pl (A.W.); marlena.gajczak@student.umed.lodz.pl (M.G.); 3 Department of Radiological and Isotopic Diagnosis and Therapy, Medical University of Lodz, Pomorska Street 251, 92-213 Lodz, Poland; szpotan_tomek@o2.pl (T.S.); agata.majos@umed.lodz.pl (A.M.)

**Keywords:** traumatic chylothorax, thoracic duct injury, blunt chest trauma, seat-belt injury, VATS, thoracic duct ligation, pleurodesis

## Abstract

Chylothorax is a rare condition caused by damage to the thoracic duct, leading to chyle from the lymphatic system leaking into the pleural cavity. The diagnosis is based on triglyceride and cholesterol levels in the pleural fluid. Various imaging methods, including computed tomography scans and lymphatic-specific investigations, may be utilized to identify the site of a chyle leak. The management of chylothorax requires a multidisciplinary approach employing conventional therapy and possibly thoraco-surgical intervention for patients who do not respond to standard treatment. Complications include malnutrition, immunosuppression, and respiratory distress. We present the case of a 57-year-old female patient who presented with chylothorax after an apparently minor car accident, illustrating problems with delayed recognition of trauma and difficulty in localizing the lymphatic leak.

## 1. Introduction

Chylothorax is a rare type of hydrothorax in which chyle accumulates in the pleural cavity. It is caused by damage to the major lymphatic vessels or the surrounding tissue [1]. Half of all cases are right-sided, 33.3% are left-sided, and 16.66% are bilateral [2]. This distribution is related to the anatomical course of the largest lymphatic vessel, the thoracic duct; damage above the fifth thoracic vertebra results in left-sided effusion, and damage below this point results in right-sided effusion [2,3]. The main role of the thoracic duct is to carry ingested fat to the circulatory system in the form of cholesterol, triglycerides, chylomicrons, and vitamins [4].

The etiology of chylothorax can be non-traumatic or traumatic. Its most common cause is a malignant tumor, most often lymphoma [4,5]; however, non-traumatic chylothorax can also result from sarcoidosis, retrosternal goiter, amyloidosis, infections (e.g., tuberculosis), benign tumors, superior vena cava thrombosis, congenital abnormalities, or diseases of the lymphatic vessels (e.g., lymphangioleiomyomatosis) [2]. Traumatic cases of chylothorax can be subclassified as non-iatrogenic or iatrogenic. Non-iatrogenic causes include penetrating wounds, blunt trauma, seat belt injuries, labor, or even forceful vomiting, sneezing, or coughing. Iatrogenic cases typically result from thoracic surgeries, especially on the esophagus [6].

There may be significant variability in the clinical presentation of chylothorax. Non-traumatic chylothorax may remain asymptomatic, especially at the initial stage when the volume of chylothorax is low. Nevertheless, the most common symptoms are breathlessness and cough [2]. In contrast, in traumatic cases, sudden injury to the thoracic duct leads to rapid accumulation of fluid in the pleural space, which may result in respiratory and hemodynamic compromise [6]. In chronic cases, chylothorax leads to loss of fat, amino acids, immunoglobulins, vitamins, electrolytes, and fluids, resulting in malnutrition, immunosuppression, pulmonary complications, and even sepsis and death [1,2,6].

The presence of fluid in body cavities can be demonstrated using various imaging techniques, including plain chest radiographs (chest X-Ray), ultrasonography, and computed tomography (CT). After a diagnosis of fluid in the pleural cavity, thoracocentesis is necessary to obtain a general assessment of the fluid. The milky appearance of pleural fluid is nonspecific [2,4]. The diagnostic criteria for chylothorax include a triglyceride (TG) level > 1.24 mmol/L (110 mg/dL) and a total cholesterol (TCH) level < 5.18 mmol/L (200 mg/dL) [2]. In case of inconclusive results—such as an intermediate pleural fluid triglyceride range of 0.56–1.24 mmol/L (50–110 mg/dL)—lipoprotein electrophoresis should be performed to confirm the presence of chylomicrons. Lipoprotein electrophoresis is an expensive test and is not readily available [7].

Radiological examinations can show increased fluid in body cavities. Chest X-Ray has limited value for identifying the specific etiology of chylothorax, except for traumatic etiology. CT of the chest, abdomen, and pelvis may narrow the diagnosis by identifying sites of traumatic injuries to the lymphatic system, compressive mediastinal or abdominal lymphadenopathy, ascites, or malignant lesions [4]. Conventional lymphangiography, CT lymphangiography, and magnetic resonance (MR) lymphangiography can also be used to visualize the lymphatic system; the selection of a particular modality is case-specific [1,4,8]. Where CT or MR imaging fails to identify the site of chyle leak, nuclear medicine imaging techniques—such as lymphoscintigraphy alone or in combination with single-photon emission computed tomography (SPECT)—may be utilized [1,4,9,10]. In addition, a biological test can be performed: the patient drinks 150 mL of a thick cream containing methylene blue or gentian violet, and the presence of the dye in the fluid confirms damage to the thoracic tube [11]. Localizing the lymphatic leak is often difficult and involves a long diagnostic process.

The initial treatment is conservative, based on pleural fluid drainage, total parenteral nutrition, talc pleurodesis, and consideration of somatostatin or a somatostatin analog, e.g., octreotide. The goal of conservative management is to decrease interstitial lymphatic flow and reduce the volume of chyle [1,2,4]. A chylothorax yielding an output of over 1 L/day, or which has persisted for longer than two weeks, will likely require a surgical procedure to achieve clinical success. Invasive procedures used to treat chylothorax include surgical thoracic duct ligation under direct visualization (video-assisted thoracoscopic surgery (VATS)/open intervention), surgical/medical pleurodesis, and a pleuroperitoneal shunt [12]. Fibrin glue or fibrin sealant, used to achieve hemostasis and seal tissues, is an effective method for improving postoperative outcomes in chylothorax. Percutaneous image-guided interventions (thoracic duct embolization and thoracic duct disruption) are used to manage patients with failed conservative therapy, high-output chylothorax, and those who cannot tolerate surgical procedures [4].

## 2. Case Presentation

A 57-year-old female patient was admitted to the Department of Internal Medicine, Diabetology and Clinical Pharmacology, Medical University of Lodz, Poland, with a nonproductive cough, dyspnea, fever up to 39 degrees Celsius, pain in the chest and abdomen, and increased abdominal circumference. The presenting symptoms had gradually increased over the two weeks before admission. The past medical history included type 2 diabetes mellitus, arterial hypertension, asthma, nodular goiter, urolithiasis, and two cysts in the pancreas. She denied any surgical procedures, trauma, or accidents. The patient was regularly receiving metformin, bisoprolol, and a combination of formoterol with beclomethasone.

On admission, the patient was alert and cooperative. Her temperature was 39 degrees Celsius. Physical examination revealed a dull percussion sound and a muffled respiratory murmur above the right lung. Saturation was 89% and 97% while breathing 2 L of oxygen, and the respiratory rate was 20 breaths per minute. Cardiac sounds were normal, but the patient presented with tachycardia: 110 beats per minute. Their blood pressure was 116/89 mmHg. The abdomen was soft and painless, with first-degree ascites. The liver was not enlarged, peristalsis was audible, and peritoneal signs were absent. Goldflam’s sign was negative on both sides, edema was absent, and peripheral lymph nodes were not enlarged.

The laboratory tests revealed the following abnormalities: a hemoglobin (HGB) level of 11.2 g/dL (reference values 12–16), a hematocrit (HCT) level of 33.6% (36–48), a platelet (PLT) level of 645 × 10^3^/µL (150–400), a white blood cell (WBC) count of 17.2 × 10^3^/µL (4.00–11.00), a neutrophil (NEU) count of 14.26 × 10^3^ µL (2.20–4.8), a monocyte (MON) count of 1.45 × 10^3^ µL (0.3–0.8), a C-reactive protein (CRP) level of 248 mg/L (0.0–5.0), an alanine aminotransferase (ALT) level of 251.4 U/L (0.0–35.0), an aspartate aminotransferase (AST) level of 158.7 U/L (0.0–35.0), a gamma-glutamyl transpeptidase (GGTP) level of 498.0 U/L (0.0–38.0), a glucose (GLU) level of 9.23 mmol/L (3.9–5.5), and presence of a bacterial urinary tract infection.

**Figure 1:**
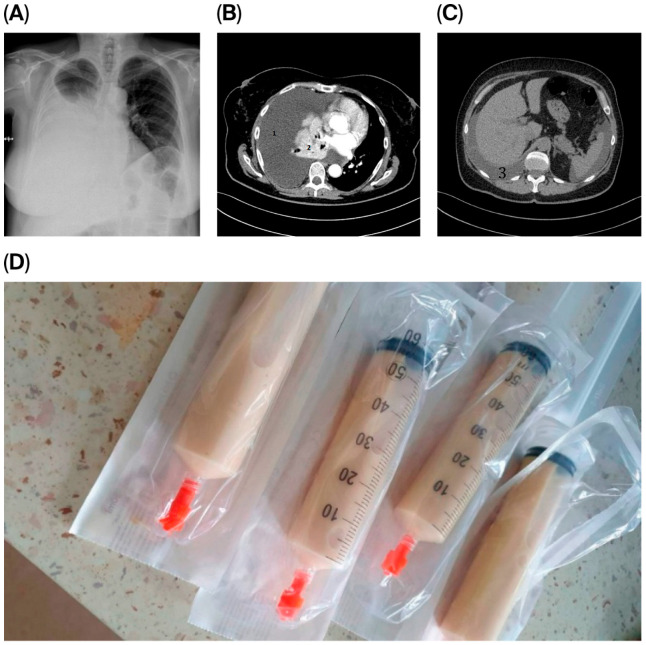
(**A**) A chest X-ray. A large amount of fluid in the right pleural cavity. (**B**) CT of the chest. Pleural effusion in the right pleural cavity (1) and atelectasis of the lower lobe of the right lung (2). (**C**) CT of the abdomen. Fluid in the peritoneal cavity next to the liver and spleen (3). (**D**) Milky fluid obtained during thoracocentesis. CT: Computed Tomography.

On the plain chest radiograph, fluid accumulation was noted in the right pleural cavity, extending to the second posterior rib (Figure 1A). Ultrasonography of the abdomen confirmed the presence of heterogeneous fluid (up to 47 mm) in the peritoneal cavity. Ultrasonography examination also revealed features of urinary retention in the left kidney (renal pelvis up to 21 mm; calyces up to 16 mm), and cysts of the left kidney (21 mm in diameter) and right ovary (22 mm in diameter). The diagnostic workup was expanded to include a CT of the chest, abdomen, and pelvis. CT of the chest confirmed a large amount of fluid in the right pleural cavity, complete atelectasis of the inferior and middle lobes, and partial atelectasis of the superior lobe (Figure 1B). CT of the abdomen confirmed fluid in the abdominal cavity (Figure 1C) and cysts in the left kidney and right ovary. A stone was found in the left kidney pelvicalyceal system, resulting in stagnation. CT did not reveal any enlarged lymph nodes, metastases, or thrombosis.

Thoracocentesis yielded 1800 mL of milky fluid (Figure 1D). The laboratory parameters met the criteria for an exudate; a TG level of 33.75 mmol/L (2989 mg/dL) and a TCH level of 4.91 mmol/L (190 mg/dL) confirmed chyle. The concentrations of pleural fluid triglycerides and total cholesterol were conclusive; therefore, lipoprotein electrophoresis was not performed. The fluid was sent for standard tests. Bacterial culture was negative; cytology showed no neoplastic cells; and the tuberculosis test was negative.

Due to pneumonia and fluid in the right pleural cavity, antibiotic therapy with ceftriaxone and levofloxacin was ordered. After confirmation of chylothorax, low-fat total parenteral nutrition by central catheter was initiated. Octreotide was considered but not used. Unfortunately, the control chest X-Rays showed a recurrence of the fluid. The patient required three thoracocentesis every two days, with evacuation of 1200–1500 mL of chyle during each procedure. After thoracic surgical consultation, drainage of the right pleural cavity was performed, and conservative treatment was continued. Further diagnostic steps were taken to find the cause of lymphorrhea and the possible site of lymph leakage. A bone marrow biopsy was performed. Lymphoma and other lymphoproliferative disorders were excluded. Lymphoscintigraphy did not localize the chyle leakage. In the control laboratory tests, the concentrations of inflammatory markers and the activity of liver enzymes were normal. Cancer markers were negative. Non-traumatic causes of chylothorax, such as malignancy, lymphoma, tuberculosis, and thrombosis, were excluded.

After several days, the patient admitted that she had been a passenger in a car accident approximately a month before hospitalization. The vehicle was moving through a city at 50–70 km/h. The woman was sitting in the front, passenger side. The patient did not require medical support at the time of the accident or hospitalization. Trauma seemed to be an identifiable and probable cause of lymphorrhea. Due to the lack of improvement after 19 days of conservative treatment, the recurrent accumulation of pleural fluid, and the likely post-traumatic etiology, the patient was reconsulted by a thoracic surgeon and deemed a candidate for surgical treatment.

The patient was transferred to the Department of Thoracic Surgery, where VATS was performed. Before surgery, the patient drank cream with methylene blue dye. During the procedure, the surgical team identified the specific site of chyle leakage into the pleural cavity, aided by the visualization of blue, milky fluid. Decortication of the right lung was performed, and the thoracic duct was dissected and ligated; the site of disruption was then reinforced with tissue glue (Glubran 2). In the postoperative period, total parenteral nutrition was applied. Due to the recurrence of lymphorrhea, pleurodesis with doxycycline was performed three times. After two weeks of hospitalization, the patient was discharged in good general condition, with a trace amount of chyle in the body cavities. During the hospital stay, the patient lost 5 kg.

We followed up with the patient for 6 months without recurrence of chylothorax. In control computed tomography of the chest, abdomen, and pelvis, the fluid was not observed. The patient did not require additional interventions. Respiratory status was good; she was without dyspnea, and her saturation without oxygen was within normal limits. The patient’s body mass returned to its initial value. The timeline of diagnostic and therapeutic process is presented in Table 1.

## 3. Discussion

There is generally a latency period of two to seven days between the time of blunt chest trauma and the onset of clinical symptoms of chylothorax, provided the injury is not a major one. However, Kakamad et al. reviewed 39 cases of chylothorax after blunt chest trauma and found that latency periods were very variable, measuring in hours, days, weeks, months, years, or even decades [13]. In our case, it is difficult to establish when chyle began to accumulate in the pleural cavity; however, symptoms were noted only one month after the injury.

Interestingly, the patient regarded the car incident as not serious, with none of the involved requiring medical support. The literature mainly includes descriptions of serious motorcycle and car accidents that cause intense chylothorax in the first hours after the accident and require immediate surgical intervention [13,14]. Comparatively, few examine the rupture of the thoracic duct caused by jerking against a seat belt [15], which was the most likely cause of post-traumatic chylothorax in the presented case. It is important to remember that any traffic accident, even an apparently minor one, can result in serious, delayed complications.

While the laboratory diagnosis of chylothorax is straightforward, identifying the site of leakage is usually a long and demanding process. Standard chest radiography and thoracic ultrasonography can effectively detect pleural effusions; however, they are unable to distinguish chylothorax from other causes of pleural effusion. CT remains a cornerstone in thoracic evaluation and is useful in identifying non-traumatic causes of chylothorax as well as detecting traumatic injuries of the lymphatic vessels. Conventional lymphangiography has been associated with many complications; therefore, it is rarely used. CT lymphangiography allows for demonstration of thoracic duct anatomy and identification of chyle leak. MR lymphangiography can also help to identify chyle leaks related to primary lymphatic disorders. Where CT or MR imaging fails to identify the site of chyle leak, nuclear medicine imaging technique may be considered [1,4,8]. Lymphoscintigraphy alone or in combination with SPECT may be utilized. The combination of lymphoscintigraphy with SPECT is more effective than lymphoscintigraphy alone for identifying anatomical structures and lymphatic leakage and hence should be recommended for the evaluation of all patients with suspected postoperative or post-traumatic chylothorax [1,4,9,10]. Unfortunately, these diagnostic methods are not widely available. Diagnostic imaging in our patient, chest radiography and CT, confirmed the presence of chyle in body cavities; however, no imaging examination, including lymphoscintigraphy, could precisely locate the site of leakage. We did not have access to lymphoscintigraphy with SPECT in our hospital.

Conservative therapy is often implemented initially because a thoracic duct leak closes spontaneously in nearly 50% of patients. This regimen includes effusion drainage (therapeutic thoracocentesis, an intercostal chest tube, or an indwelling pleural catheter), nutritional adjustments (such as a medium-chain triglyceride diet or total parenteral nutrition), and pharmacotherapy (somatostatin or its analogs) [1,2,3,5].

Several authors suggest categorizing cases into high-output and low-output chylothorax based on volume. High-output chylothorax involves an estimated or confirmed fluid loss exceeding 1 L daily, whereas low-output chylothorax refers to a daily volume below 1 L [1]. Some researchers recommend surgical intervention for a daily output exceeding 1 L/24 h or persistent leakage lasting more than two weeks; others propose a threshold of drainage > 1.5 L/24 h, an output of 1 L/24 h sustained for five days, or the development of nutrition/metabolic compromise [16]. It is also important to consider the underlying cause of chylothorax. Low-output chylothorax occurs more frequently in non-traumatic etiologies, while high-output cases are more common found in post-surgical and post-traumatic patients. In non-traumatic chylothorax, there is no strong indication for immediate surgical intervention. In contrast, post-traumatic or post-surgical chylothorax requires aggressive early surgical intervention [2]. In our patient, the delayed recognition of trauma influenced the thoracic surgeons’ initial decision to pursue conservative treatment while simultaneously investigating the etiology of the chylothorax.

Invasive procedures used to treat chylothorax include surgical thoracic duct ligation under direct visualization (video-assisted thoracoscopic surgery (VATS)/open intervention), surgical/medical pleurodesis, and a pleuroperitoneal shunt. The most common surgical procedure is thoracic duct ligation. To improve intraoperative visibility and isolate the precise location of the lymphatic leak, specialized techniques may be utilized, such as the administration of specific liquids or the use of lipophilic dyes like indocyanine green to assist in visualization. This surgical technique is highly successful in traumatic cases, boasting efficacy rates over 90%. In non-traumatic populations, therapeutic outcomes remain less established. For persistent, treatment-resistant conditions, specialized shunting operations may be utilized as a salvage therapy, despite their correlation with elevated complication rates [1,5,12].

Percutaneous image-guided interventions (thoracic duct embolization and thoracic duct disruption) provide an alternative therapeutic pathway. These interventions are particularly indicated for individuals presenting with high-output chylothorax, as well as those whose clinical status precludes standard open or thoracoscopic surgery [4,6].

Furthermore, there are currently no guidelines regarding the management of chylothorax. High-quality evidence, including randomized controlled trials, is lacking, so management approaches are largely based on clinical consensus, case series data, and observational studies. A multidisciplinary approach is crucial, involving internal medicine specialists and thoracic surgeons [17].

## 4. Conclusions

Chylothorax is a rare but very serious condition. Laboratory diagnosis of pleural effusion is relatively easy based on the concentration of TG and TCH in the pleural fluid, but identifying the site of leakage is often difficult and demanding. We demonstrated the problems with delayed recognition of trauma after an apparently minor car accident. We would like to emphasize the need for repeated and detailed history-taking after trauma to establish the right diagnosis. Early multidisciplinary escalation is needed when conservative therapy fails.

## Figures and Tables

**Table 1:** Timeline of case report.

16 July	Car accident
16 August	Admission to the hospital with symptoms of pneumonia.
16 August	X-Ray of chest, ultrasonography of abdomen, and confirmation of fluid in the right pleural cavity.
18 August	Thoracentesis and evacuation of 1500 mL of fluid, diagnosis of chylothorax, and initiation of conservative therapy.
22 August	CT scan of chest, abdomen, and pelvis.
22 August	Thoracentesis and evacuation of 1200 mL of chyle.
29 August	Thoracentesis and evacuation of 1500 mL of chyle.
2 September	Drainage of the right pleural cavity and evacuation of 1200 mL of fluid.
Following days	Evacuation of 1200–1500 mL of chyle every day.
5 September	The patient admitted that she had been a passenger in a car accident.
6 September	Qualifications for hospitalization in the Department of Thoracic Surgery.
7 September	Drainage of the right pleural cavity and evacuation of 1200 mL of fluid.
9 September	VATS, the right lung was decorticated, the thoracic duct was dissected, ligated, and the break was sealed with tissue glue.
next days	Three pleurodesis with doxycycline.
21 September	Discharge from the hospital.

## Data Availability

The original contributions presented in this study are included in the article. Further inquiries can be directed to the corresponding author.
